# Aged Biogenic Carbonates from Crustacean Waste: Structural and Functional Evaluation of Calibrated Fine Powders and Their Conversion into Phosphate Minerals

**DOI:** 10.3390/ma18225119

**Published:** 2025-11-11

**Authors:** Ilirjana Bajama, Karlo Maškarić, Geza Lazar, Tudor Tamaş, Codruţ Costinaş, Lucian Barbu-Tudoran, Simona Cîntă Pinzaru

**Affiliations:** 1 Faculty of Physics, Babes-Bolyai University, Kogalniceanu 1, 400084 Cluj-Napoca, Romania; karlo.maskaric@ubbcluj.ro (K.M.); geza.lazar@ubbcluj.ro (G.L.); codrut.costinas@ubbcluj.ro (C.C.); 2 Institute for Research, Development and Innovation in Applied Natural Science, Babes-Bolyai University, Fântânele 30, 400327 Cluj-Napoca, Romania; 3 Advanced Research and Technology Center for Alternative Energy, National Institute for Research and Development of Isotopic and Molecular Technologies, Donath 67-103, 400293 Cluj-Napoca, Romania; 4 Faculty of Biology and Geology, Babes-Bolyai University, Kogalniceanu 1, 400084 Cluj-Napoca, Romania; tudor.tamas@ubbcluj.ro (T.T.); lucian.barbu@ubbcluj.ro (L.B.-T.); 5 Integrated Laboratory of Electron Microscopy, National Institute for Research and Development of Isotopic and Molecular Technologies, Donath 67-103, 400293 Cluj-Napoca, Romania

**Keywords:** aged biogenic waste, antioxidant preservation, calibrated powders, ball milling, Raman, XRD, IR, SEM-EDX

## Abstract

Seafood-derived carbonate waste, primarily calcium carbonate (CaCO_3_), has attracted growing interest for sustainable reuse, yet the unique potential of aged biogenic sources remains underexplored. Blue crab (*Callinectes sapidus*) shells are particularly distinctive: they consist of Mg-calcite with an intrinsic 3D-porous structure and naturally embedded astaxanthin, a potent antioxidant not found in other calcite- or aragonite-based residues. While organic degradation over time is often assumed to compromise functionality, this study demonstrates that five-years-aged crustacean shell waste retains both its crystallinity and bioactive carotenoids after calibrated ball milling. Across four powder batches produced under distinct milling conditions by varying frequencies and durations, dynamic light scattering confirmed only subtle particle size variation, while Raman spectroscopy, XRD, FT-IR, and SEM-EDX confirmed structural and chemical integrity and highlighted the subtle amorphization induced by slightly different milling parameters, which, in turn, driven to slightly different conversion efficiency into phosphate mineral. Strikingly, all powders underwent rapid transformation into dicalcium phosphate dihydrate (brushite) enriched with carotenoids upon reaction with phosphoric acid. This work reveals, for the first time, that years-aged biogenic Mg-calcite waste not only preserves its naturally embedded carotenoids but also offers a direct route to functional phosphate composites, establishing its untapped value in environmental and biomedical applications.

## 1. Introduction

The management and valorization of marine shell waste have become increasingly important within sustainable resource recovery and circular economy frameworks. Each year, large quantities of crustacean and mollusk exoskeletons are discarded as by-products of seafood consumption and processing, creating significant disposal and environmental challenges in coastal areas [1,2,3]. These residues are mainly composed of biogenic calcium carbonate, bound within an organic matrix of chitin, proteins, and pigments, and therefore represent a renewable yet underexploited raw material. Developing efficient strategies to repurpose such residues contributes to both waste reduction and the production of functional bio-derived materials for industrial and environmental applications [4,5,6].

Among these residues, the shells represent a notable example of seafood by-products generated from commercial processing. When accumulated and stored over long periods, such shells undergo gradual organic degradation and mineral changes, forming aged organic–mineral detritus rich in calcium carbonate and residual pigments. Although often considered waste, these materials retain valuable biogenic mineral structures that can be re-engineered into functional compounds. Recent research has demonstrated the potential of marine biogenic carbonates as precursors for calcium phosphate materials, which have applications in catalysis, environmental remediation, and biomaterials [7,8,9].

The present study comprehensively reports the composition, morphology, and reconversion of years-aged, post-consumption blue crab (*Callinectes sapidus*) shell waste biomaterial milled to powder, which initially has been exposed to thermal treatment (cooked). The blue shells turned red via thermal processing along the seafood chain, and the post-consumption leftover shells were preserved as waste for a long time (5 years). This situation resembles well the current management of seafood waste as land filled deposits along many seashores. Thus, the aged biogenic waste was subject to calibrated ball milling to powders, analyzed regarding their eventually altered composition, size-distribution, and morphology, and finally converted into phosphate minerals under phosphoric acid exposure. The work focuses on the structural preservation and reactivity of the aged material and evaluates how controlled milling influences particle properties and conversion efficiency. This approach provides a model for valorizing long-stored marine shell waste and can be extended to other crustacean and mollusk residues worldwide, contributing to circular economy strategies for the sustainable reuse of marine biomineral resources.

Biogenic carbonates derived from marine organisms have gained increasing attention due to their potential across biomedical, pharmaceutical, environmental, industrial, and agricultural applications [10,11,12,13,14,15,16,17,18,19]. These materials offer a sustainable alternative for resource recovery and advanced material synthesis. The ability to control particle size and surface properties through milling, while preserving structural features of the organic-inorganic framework, supports their reuse in environmental remediation and material development [14,15,16,18,19,20,21,22,23,24,25,26].

Biogenic calcium carbonate occurs in several polymorphic forms—including calcite, aragonite, vaterite, and amorphous calcium carbonate (ACC)—with polymorphism strongly linked to species-specific [18] biogenesis. Crustacean-derived biogenic carbonate displays highly porous, hierarchically structured architectures composed of calcium carbonate and embedded organic materials like chitin, proteins, and pigments [10,15,16,21,22]. These features enabled its use for new pharmaceutical excipients, new biostimulants [26], active adsorbents [22], for controlled release of active ingredients [24,25], and other applications, particularly in the removal of heavy metals, dyes, and pharmaceuticals from waste waters [10,11,13,14,17].

Ball milling enhances the adsorption potential of biogenic carbonates by reducing particle size, increasing surface area, and improving dispersion [8,18,19,20,27]. However, excessive milling can induce phase transitions or amorphization, leading to a loss of crystalline order and potentially decreasing functional performance [9,18,19,21,22]. Optimizing milling conditions is therefore critical for maintaining the balance between surface enhancement and structural stability [9,18,20].

Characterization techniques such as Raman spectroscopy, FT-IR, and X-ray diffraction (XRD) are vital for understanding these structural modifications [15,21]. Raman spectroscopy provides detailed vibrational data on molecular bonding and lattice distortion [16,21,22], while FT-IR complementarily detects changes in functional groups and residual organics [15,22]. XRD identifies crystallographic transitions and quantifies crystallinity, which is important in evaluating the effects of milling and thermal processing [15].

Thermal treatments during food preparation, such as boiling or steaming of crustaceans, can degrade organic components and alter the physical characteristics of shells [14,15,16]. Cooking may promote the release of astaxanthin from crustacyanin complexes, changing the visual pigmentation and chemical makeup [16]. Aging of cooked shell waste further impacts the material through slow degradation of organics, presenting a realistic model of landfilled biowaste [11,15]. Understanding these transformations is essential for assessing the reusability of long-stored waste materials.

Moreover, biogenic carbonates from post-extraction of carotenoids increased porosity, with the specific surface area from less than 10 m^2^/g in native shells to over 30 m^2^/g in treated powders [21,26]. These features are particularly relevant for adsorption or catalytic applications, where active surface area is a limiting factor [16,22].

Biogenic carbonates have also been successfully employed as carriers in pharmaceutical applications. Drug delivery systems using 5-fluorouracil-loaded nanoporous biogenic calcite showed controlled release behaviors and pH responsiveness [24,25]. Moreover, milling-assisted processing has enabled their integration into biofertilizers and polymer matrices for sustainable material development in various fields [26,28].

The conversion of milled biogenic carbonates into phosphate minerals (e.g., brushite, hydroxyapatites) using phosphoric acid further extends their biomedical potential [20,29,30,31,32], which is advantageous in bone tissue engineering and regenerative applications [29,30,31,32]. The use of crab shell waste as a phosphate precursor aligns with circular economy principles by transforming food waste into high-value biomaterials.

This study explores how various ball milling conditions (frequency, time) influence the physicochemical properties of powders from years-aged biogenic waste from crustaceans and their re-purpose for synthesizing green phosphate minerals. In the reported studies so far, aged crustacean biowaste has never been addressed before. Here, using Raman, FT-IR, and XRD analysis, along with SEM and porosity studies, we investigate the structural, morphological, and functional evolution of these materials to determine optimal processing routes that retain or enhance functional performance while enabling sustainable reuse of long-term stored marine biowaste. Through a holistic approach combining mechanical processing and spectroscopic analysis, this work supports the development of eco-friendly, functional materials from aged, discarded crustacean waste for their knowledge-based valorization, environmental remediation, and green, new material syntheses in alignment with blue bioeconomy and circular sustainability goals. We further showed how controlled milling conditions subtly determine the structure and morphology of the phosphate mineral product.

## 2. Materials and Methods

### 2.1. Biogenic Carbonate Waste Material

**Figure 1:**
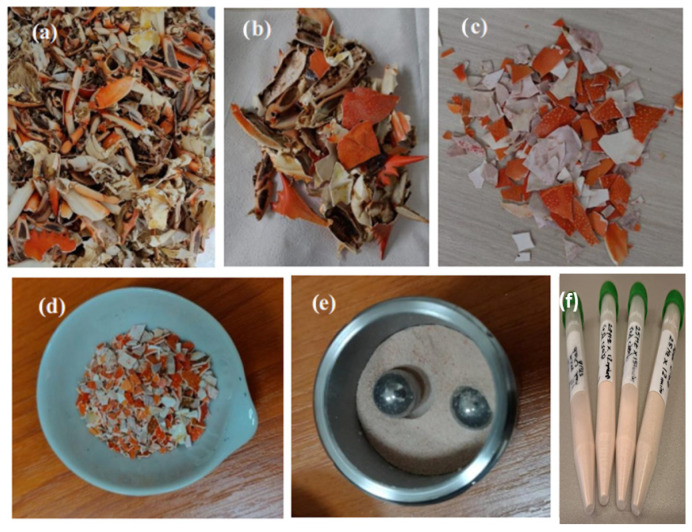
Images of the sample appearance during the steps performed for processing the raw material, represented by the aged biogenic waste: (**a**,**b**) cooked crab shell before and after selecting suitable fragments; (**c**) cleaned raw material; (**d**) cleaned and mechanically crushed material; (**e**) biogenic powder obtained through the ball milling process; (**f**) obtained powder samples.

Here, five-years-aged biogenic waste carapace originating from an initial waste material stock preserved in cold and dark conditions, collected from the Adriatic coast, Croatia [15] has been employed. The shells were cleaned of self-detachable dried organic membrane, washed and dried, and one large stock of ground material has been divided into four identical sub-stocks, relevant for calibrated ball-milling under controlled conditions, as shown in Figure 1. The primary material was first manually crushed into smaller fragments to facilitate further ball milling (Figure 1c,d).

### 2.2. Ball Milling

The fragmented shells were then processed into fine powder using a Retsch Mixer Mill MM 400 (Retsch GmbH, Haan, Germany) ball mill, an automatic system with controlled milling time and frequency, to ensure the powders’ reproducibility. Milling was carried out in stainless-steel jars (50 mL) with stainless-steel balls (10 mm diameter). The procedure employed six grinding balls, with parameters set at a frequency of 22 and 25 Hz and duration of 12 and 15 min, resulting in four groups of samples, at 25 Hz for 12 min, 25 Hz for 15 min, 27 Hz for 12 min, and 27 Hz for 15 min, respectively. The milling parameters (25–27 Hz, 12–15 min) were selected considering the limitations of the ball milling domain of frequency (1–30 Hz), considering that low values are not effective for milling. Thus, the values of 25 and 27 Hz were considered relevant for the equipment capability and its milling calibration performance. Milling time difference is 25% increased (from 12 to 15 min), and it was considered relevant for the lab conditions, because a lower time was not practical for powdering, while a higher time is usually inconvenient for repetitive tasks in high quantities. Particular attention was given to minimizing contamination from the stainless-steel milling components. Thus, we chose two values for frequency and time within the recommended exploitation regime, namely two different values below the maximum frequency (<30 Hz), namely 25 and 27 Hz, and two time settings in minutes (12 and 15), according to the feasible workload for repetitive sample sets, to be processed at once and repeatedly milled replicas. Thus, we changed the time from 12 to 15 min to have all the samples and their replicas within the same date, and thus, to avoid additional bias in considering waste materials at different times.

### 2.3. Micro-Raman Spectroscopy

A Renishaw InVia Reflex Raman system (Renishaw plc, Gloucestershire, UK) with a Leica microscope and an ultra-fast Centrus detector with high sensitivity and speed has been employed. For Raman excitation, a Cobolt diode-pumped solid-state laser (Cobolt AB, Solna, Sweden) operating at 532 nm was used. Dozens of spectra have been collected from individual powder particles from each group, using the 5× (NA 0.12, WD 13.2 mm), 20× (NA 0.35, WD 2 mm), and 100× (NA 0.9, WD 3.4 mm) microscope objectives. An edge filter has been employed to record spectra in the 50−1846 cm^−1^ spectral range with 0.5 cm^−1^ resolution. The laser power and acquisition parameters were optimized in the Wire 5.6 software to ensure a reasonable signal-to-background while simultaneously recording information from mineral and organic phases of biogenic powders. Higher laser power induced a higher background due to the high fluorescence signal of the organic counterparts of the particles. Micrographs of the powders have been taken prior to Raman measurements using the Wire 5.6 system video camera.

### 2.4. Fourier Transform Infrared Spectroscopy (FT-IR)

A Shimadzu IR Spirit QATR-S2 FT-IR spectrometer (Shimadzu Corporation, Kyoto, Japan) has been employed for powder characterization. Spectra were collected using 50 scans, in the range of 400–4000 cm^−1^, to confirm the presence of characteristic functional groups such as amides (from chitin), carbonates (from calcium carbonate), and hydroxyl groups, providing insights into the organic and inorganic components of the aged, powdered material.

### 2.5. X-Ray Diffraction (XRD)

XRD data have been recorded using a Bruker D8 Advance (Bruker Company, Karlsruhe, Germany) diffractometer in Bragg–Brentano geometry with a Cu tube with λk_α_ = 0.15418 nm, Ni filter, and a LynxEye detector (Bruker Corporation, Austin, TX, USA). Corundum (NIST SRM1976a) was used as an internal standard. The data were collected over the 3.8–64° 2θ interval at a 0.02° 2θ step, with 0.5 s per step. The mineral identification has been performed using the Diffrac.Eva 2.1 software with the PDF2 (release 2023) database.

### 2.6. Dynamic Light Scattering (DLS)

A Malvern Zetasizer Nano ZS90 particle analyzer (Malvern Instruments Ltd., Worcestershire, UK) equipped with a He-Ne laser (Lumentum Operations LLC, Sunnyvale, CA, USA) (633 nm, 5 mW) was used to examine particle size distributions, dissolved in ethanol absolute. Each investigation consisted of three sets of 15 measurements, which were averaged, all taken at a scattering angle of 90° and at a temperature of 25 °C. The laser attenuation level for each measurement was selected automatically by the software. All samples have been sonicated for 90 min at 25 °C before measuring.

### 2.7. SEM-EDX

Scanning electron microscopy (SEM) coupled with energy dispersive spectroscopy (EDS) was effectuated with a Hitachi SU8230 (Hitachi Company, Tokyo, Japan) equipped with an X-Max 1160 EDX element detector (Oxford Instruments, Oxford, UK). Images were taken at an acceleration voltage of 30 kV. The sample’s surface was sputtered with a gold thin film of 9 nm prior to analysis, to enhance surface conductivity and minimize charging effects under the electron beam. SEM imaging enabled detailed visualization of the powder morphology, revealing particle shape, surface texture, and aggregation states. The powders exhibited irregular granular structures, with broad size distribution influenced by milling conditions, and the EDS mapping of the sample area provided a useful, semiquantitative evaluation of the main elemental distribution in powders.

### 2.8. Conversion of Biogenic Powders to Phosphate Minerals

Equal amounts of 0.3 g from each powder biomaterial were mixed with 600 µL of orthophosphoric acid 25% (PanReac AppliChem, ITW Reagents, Barcelona, Spain), respectively, under constant stirring at room temperature.

The four powder samples treatment with phosphoric acid has been achieved based on the stoichiometry for hydroxyapatite conversion, as previously described in our recent study [32] using the drop-by-drop acid addition to avoid excessive bubbling during effervescent reaction and to prevent ingredients from pouring. In that study, we developed and optimized a sustainable route that transforms biogenic calcite into various calcium phosphate phases, and, secondly, we demonstrated that Raman technology with portable instruments served as an effective, real-time, in situ analytical tool for monitoring the conversion process towards the desired phosphate mineral species [32].

The resulting reaction products from aged material were dried at room temperature and further analyzed.

## 3. Results and Discussion

### 3.1. Aged Biogenic Powders: Structure, Morphology, and Particle Size Distribution

Given the aged waste material, its evaluation has been conducted using primary micro-Raman spectroscopy combined with optical microscopy in conjunction with its characterization using XRD, FT-IR, and DLS.

#### 3.1.1. Optical Microscopy of Powder Particles

**Figure 2:**
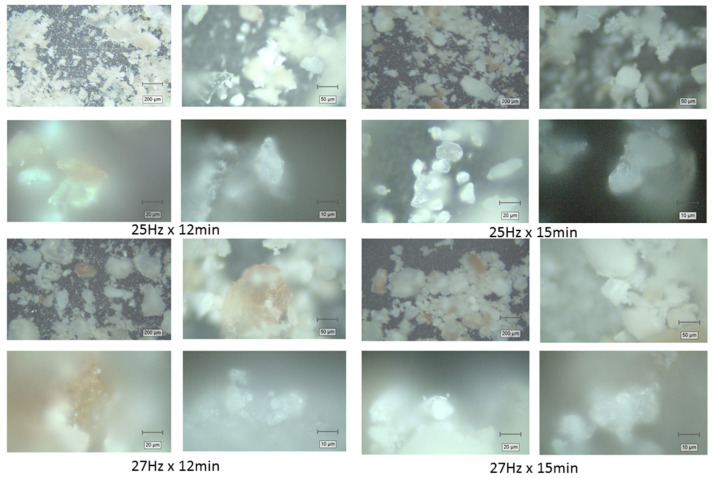
Representative optical microscopy images taken with the video camera of the Raman microscope prior to measurements, showing the morphology of the aged biogenic powder obtained under milling conditions 25 Hz for 12 min, 25 Hz for 15 min, 27 Hz for 12 min, and 27 Hz for 15 min, at 5×, 20×, 50×, and 100× magnification objectives, respectively, in the 4 × 4 groups, as indicated.

To evaluate the morphology and particle size distribution of the crab shell powders, microscopic images were captured using a Raman spectrometer at magnifications of 5×, 20×, 50×, and 100× for each powder sample prior to Raman analysis. The four powders obtained under different milling conditions (25 Hz for 12 min, 25 Hz for 15 min, 27 Hz for 12 min, and 27 Hz for 15 min) were analyzed to assess the impact of milling parameters on particle size and uniformity. The average particle sizes optically observed at each magnification for each powder sample are summarized in Table 1, and their appearance is illustrated in Figure 2. The powder particles appeared bright white or pinkish brown, indicating an inhomogeneous pigmentation, as expected.

Surprisingly, despite the variations in milling frequency and duration, no significant difference was optically observed in the particle sizes across the different conditions. At all magnifications (5×, 20×, 50×, and 100×), the powders exhibited similar morphology, with irregularly shaped particles and comparable, broad size distributions from about 1–2 μm to hundreds of micrometers, as illustrated in Figure 2. The images from the different samples showed that the particles were optically rather similar, with small, bright white fragments of calcite crystals mixed with larger, stronger pigmented fragments. The slightly changed milling conditions were poorly reflected in the optical microscopy appearance of biogenic powders.

#### 3.1.2. X-Ray Diffraction (XRD) Analysis of Aged Powders

**Figure 3:**
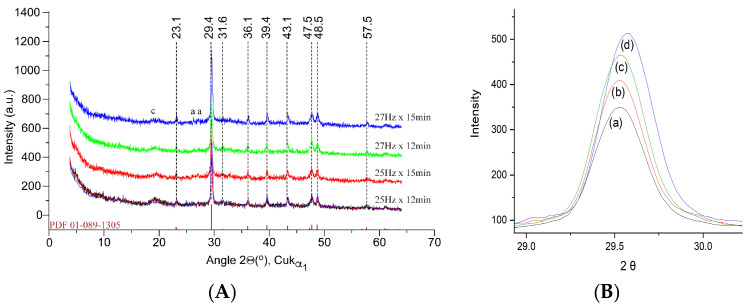
(**A**) X-ray diffractograms of the biogenic powders milled under different conditions versus the magnesium calcite pattern from the 01-089-1305 PDF file [30]. Minor peaks of aragonite (a) and chitin (c) also occurred. (**B**) Details of the main calcite band at a 29.4 angle are given to highlight the subtle band profile change with milling conditions. Spectra (**a**–**d**) correspond to samples milled at 25 Hz × 12 min, 25 Hz × 15 min, 27 Hz × 12 min, and 27 Hz × 15 min, respectively.

X-ray diffraction (XRD) was employed to investigate the crystallographic structure of the crab shell powders under different milling conditions. The XRD patterns (Figure 3) were obtained for all four powder samples to assess the impact of milling on the crystallinity and phase composition of the powders. They showed prominent peaks corresponding to calcium carbonate (CaCO_3_), specifically the calcite phase, which is the primary mineral component of the fresh crab shell. The characteristic diffraction peaks of calcite were observed at 2θ values of approximately 23.1°, 29.4°, 31.6°, 36.1°, 39.4°, 43.1°, 47.5°, 48.5°, and 57.5°, consistent with a common XRD pattern of magnesium calcite [32], as well as with previous results obtained on mixed cuticle samples with variable crystallinity [16], with no discernible effect of milling conditions on the overall patterns recorded. Minor reflections of aragonite denoted “a” in Figure 3, at 26.2° and 27.2°, as well as a broad peak characteristic of chitin (denoted “c” and centered at 19.5°), were also recorded.

#### 3.1.3. Fourier Transform Infrared Spectroscopy (FT-IR) Analysis of Powders

**Figure 4:**
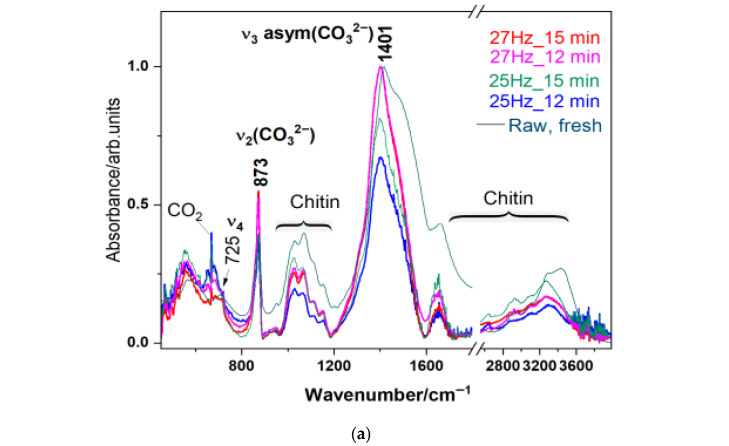

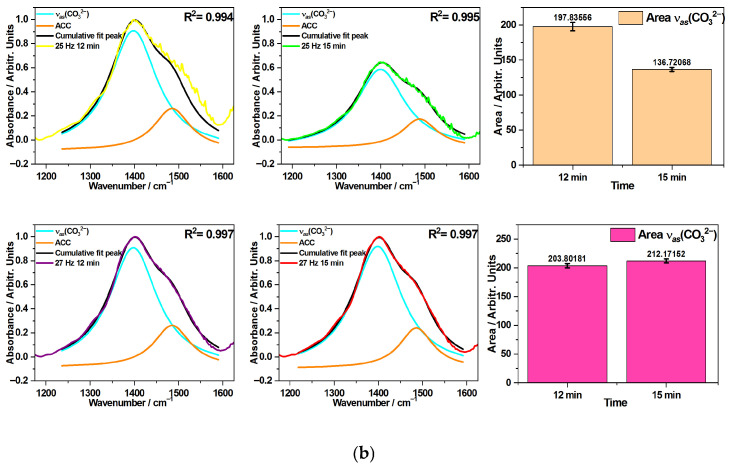
(**a**) Overlay of FT-IR spectra of biogenic powders milled under different conditions: 25 Hz for 12 min, 25 Hz for 15 min, 27 Hz for 12 min, and 27 Hz for 15 min, as indicated by color codes. The main carbonate bands are plotted. A FT-IR spectrum of the raw, fresh biogenic material (blue crab carapace) is shown for comparison. (**b**) Deconvoluted IR band in the spectral range from 1200 to 1600 cm^−1^ comprising the main band assigned to the υ_3_ asym (CO_3_^2−^) mode peaking at 1401 cm^−1^, (red line) and the amorphous calcium carbonate ACC (green line), changing in band features depending on time and frequency of ball milling. The comparative plot of the band area of the ν_3_ asym (CO_3_^2−^) mode for two distinct milling times, under 25 Hz and 27 Hz frequencies, respectively, is given on the right side. The highest band area was recorded for 27 Hz_15 min.

FT-IR spectroscopy was employed to analyze the functional groups and chemical bonds characteristic of biogenic waste powders under four different milling conditions. FT-IR spectra were recorded to assess any changes in the molecular structure and surface chemistry induced by the milling process. The FT-IR spectra for all four crab shell powders (Figure 4) exhibited characteristic absorption bands corresponding to the main functional groups typically found in calcium carbonate (CaCO_3_) polymorphs calcite and ACC, respectively, where the ACC exhibits a more prominent shoulder at 1483 cm^−1^, which is overlapped with characteristic band of the organic components such as proteins and chitin, present in the crab shells.

The FT-IR spectra of all four crab shell powders exhibited characteristic absorption bands corresponding to the main functional groups found in calcium carbonate (CaCO_3_) and organic components such as proteins and chitin [15]. The most prominent peaks observed were at 1401 cm^−1^, corresponding to the asymmetric stretching vibration of the carbonate (CO_3_^2−^) group, and 873 cm^−1^, attributed to the out-of-plane bending vibration of the same group, confirming the presence of the calcite phase preservation in aged biogenic material. A multi-component broad absorption band in the 3600–3200 cm^−1^ region was associated with O-H stretching vibrations, likely due to surface hydroxyl groups or adsorbed moisture. Additionally, the peak at 1650 cm^−1^ is primarily associated with chitin but could also be linked to C=O stretching vibrations from amide I groups, suggesting the presence of organic compounds, likely residual proteins within the powder. The spectra remained consistent across all milling conditions, with subtle changes in the area of the characteristic modes of carbonate, suggesting that the milling process did not induce chemical modifications or degradation of the material but slightly influenced the crystalline–amorphous balance. The sharpest peaks were observed for the 27 Hz_15 min milling conditions. Therefore, the asymmetric stretching mode of carbonate was used for comparison. This band has been Lorentzian deconvoluted to reveal the main component at 1401 cm^−1^ and an additional one at 1483 cm^−1^, the latter being associated with the amorphous calcium carbonate (ACC). These comparative data are illustrated in Figure 4b, which clearly shows that the highest carbonate band area was recorded for the highest time and highest frequency (27 Hx_15 min), while the lowest was for the 25 Hz_12 min. These results confirm that the structural integrity of both the inorganic and organic components in the aged crab shell powder was preserved, with slight changes in the crystalline–amorphous ratio as a function of ball milling conditions. This slight change suggests that functional properties remain mostly intact while benefiting from increased surface area.

#### 3.1.4. Micro-Raman Spectroscopy of Aged Powders

**Figure 5:**
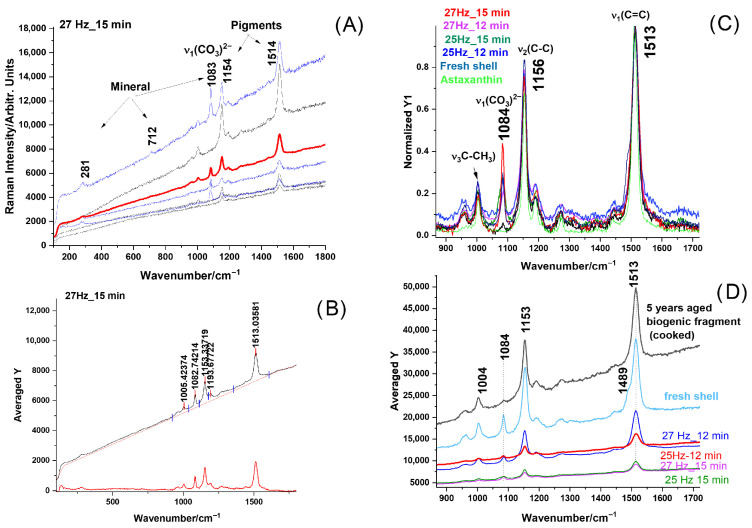
Micro-Raman spectra excited with 532 nm of the four powders resulted from the milling the aged biogenic material under different conditions: (**A**) typical raw spectra collected from five distinct powder particles from the finest milled sample (27 Hz for 15 min) and their averaged (red line) spectrum; particles with strong resonance Raman signal of carotenoids (black lines) and particles with stronger calcite bands (blue line) are observed; (**B**) raw Raman signal of random powder particle showing both the calcite and astaxanthin pigment bands, as indicated, and the corresponding background subtracted signal; (**C**) comparative, background-subtracted, and normalized averaged Raman signal of the four powders plotted in color codes as indicated, for milling at 25 Hz for 12 min, 25 Hz for 15 min, 27 Hz for 12 min, and 27 Hz for 15 min, respectively, compared to the RR signal of pure astaxanthin (green line) and with signal of fresh biogenic material (light blue line). (**D**) A zoomed-in view of the 900–1700 cm^−1^ range of the averaged, raw spectra of the four powders, compared with the signal from an aged shell fragment (not milled) and one fresh fragment (light blue line), suggests a slight broadening in the carotenoid profile band in aged fragments or powders.

Raman spectroscopy was utilized to examine the structural composition and vibrational characteristics of powder particles processed under the four different milling conditions, aiming to assess the status of both the mineral and native pigments and their possible changes during waste aging and subsequent milling. Examples of raw Raman data of powder particles, the procedure to compare them among each other, and, further, to compare the aged material with the fresh biogenic material are shown in Figure 5. Micro-Raman spectra excited with 532 nm of the four powders resulted from the aged biogenic material milling under different conditions, shown in each sample of three distinct cases: (i) typical powder particles exhibiting only mineral bands, (ii) particles with pigment bands only and a strong fluorescence background completely covering the mineral signal, and (iii) particles with both mineral and pigment bands. Such examples are illustrated in Figure 5A, collected from the finest milled sample (27 Hz for 15 min). This inhomogeneity is not surprising, since we showed earlier that the pigments are abundantly distributed mainly in the exocuticle of the native crustacean [16]. In native, fresh shells, carotenoid content, which is proportional to the intensity of the v_1_ Raman band of the C=C stretching mode, displays a variable distribution in exocuticle, peaking at a maximum at about 10 to 20 μm below the cuticle outer surface [16]. Thus, powder particles bearing high pigment content have clearly resulted from the exocuticle layer of the fragments.

Surprisingly, all the powders showed preserved carotenoids, as shown in Figure 5C. Moreover, their peak at about 1513 cm^−1^ is consistent with the carotenoid band in fresh shells or in aged fragments, indicating that the natural degradation of the organic counterpart, accompanied by the characteristic volatile compounds generating a specific smell, did not affect the carotenoids. Their preservation in such intricated biomaterial during aging might raise additional added value to these materials, as naturally embedded with powerful astaxanthin antioxidant preserved after years of aging. These findings might open additional biomedical applications.

The pigment is primarily composed of astaxanthin (the main carotenoid), as a carotenoid-protein breakage when the blue shell is exposed to high temperature (cooked crab shells). Thus, the pink-reddish color is widely observed in freshly cooked blue crabs. As mentioned in the Introduction, according to previous studies [21], such pigments can be further extracted or recovered for potential applications in natural colorants and antioxidant formulations. This addition strengthens the context of pigment preservation and its possible valorization. The presence of pigment, known for its strong antioxidant properties, highlights the unique advantage of obtaining, via a sustainable route, a phosphate mineral with potentially embedded carotenoid, if not destroyed under the conversion reaction.

Data processing for comparative analyses of the powders implied collecting dozens of micro-Raman spectra of fine particles from each stock and calculating their average Raman signal, as indicated in Figure 5B. Their average (red line) spectrum was further taken for comparison among powder stocks. Particles with a strong resonance Raman signal of carotenoids (black lines) and particles with stronger calcite bands (blue line) were observed in all four stocks. The background-subtracted signal of the averaged spectra is comparatively displayed in Figure 5C, after normalization to a unit, for the four powders plotted in color codes as indicated, for milling at 25 Hz for 12 min, 25 Hz for 15 min, 27 Hz for 12 min, and 27 Hz for 15 min, respectively. The signal was compared to the RR signal of pure astaxanthin (green line) and to the signal of fresh biogenic material (light blue line). As shown in Figure 5D, a zoomed-in view of the 900–1700 cm^−1^ range of the averaged, raw spectra of the four powders, compared with the signal from an aged shell fragment (not milled) and one fresh fragment (light blue line), suggested a slight broadening in the carotenoid profile band in aged fragments or powders.

#### 3.1.5. Dynamic Light Scattering (DLS) Analysis

**Figure 6:**
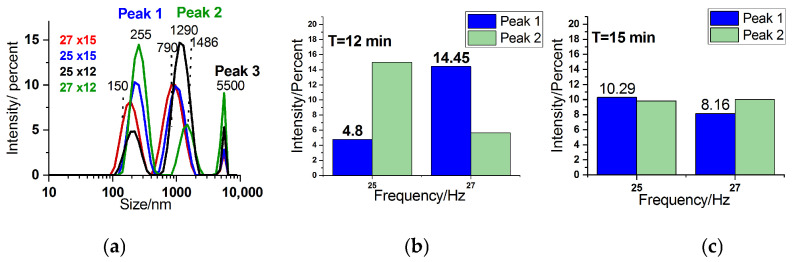
Dynamic light scattering (DLS) analysis of crab shell powders milled under various conditions. (**a**) Overlay of DLS spectra for all samples milled under different conditions: 27 Hz for 15 min, 25 Hz for 15 min, 25 Hz for 12 min, 27 Hz for 12 min. (**b**) Column plot showing the dependence of peak intensity on frequency for a milling time of 12 min and (**c**) 15 min.

The particle size distribution of the crab shell samples was analyzed using dynamic light scattering (DLS). Basically, the preliminary DLS data showed that the distribution is affected by the sedimentation behavior of the particles and the large size distribution present within the samples. No additional pre-treatment, such as centrifugation or filtration, was applied to the dispersions prior to DLS analysis. This was performed deliberately to preserve the authentic particle size distribution of the ball-milled powders, including both fine and coarse fractions. Although pre-treatment could narrow the measured distribution, our aim was to capture the entire polydisperse population produced by ball milling. DLS analysis (Figure 6) revealed two primary peaks in the size distribution, one at approximately 255 nm and another at around 1290 nm. DLS algorithms attempt to fit the autocorrelation data into a limited number of discrete peaks, often oversimplifying the actual size distribution and failing to capture the full complexity of the sample. This is especially problematic when the particle population spans a broad range, from hundreds to several thousand nanometers, as the inversion algorithms are not well-suited for resolving continuous or polydisperse distributions. In our case, for example, the DLS output consistently shows distinct peaks around 255 nm and 1290 nm, with no other peaks in between, even though we believe that there is no reason for intermediate-sized particles not to exist. This gap is likely an artifact of the fitting process, where the algorithm resolves only the dominant modes it can detect with confidence, disregarding less well-defined or overlapping size contributions. A third peak cut off at about 5500 nm suggests large particles that cannot stay within the dispersion and produce sedimentation.

Within the peak intensity distribution for the two different milling times and frequencies (Figure 6b,c), the third peak was cut off due to limitations in the software, which is programmed to calculate the distribution with a bias toward smaller particles. As a result, larger particles beyond 5000 nm could exist in the sample. The software has limitations when measuring large particle size distribution simultaneously in the sub-micrometer range and those larger than 5000 nm, leading to truncation of the size distribution at this point. To provide a relative comparison, the intensity of the 5000 nm peak could be used as an indicator of the number of larger particles present in the sample. A higher intensity in this region suggests a greater abundance of very large particles.

Overall, while the DLS analysis offers valuable insight into the size distribution of the biogenic particles, the limitations of the measurement technique and software may influence the accuracy, particularly for larger particles beyond the 5000 nm cutoff.

Given the results, the size distribution found with DLS in the sub-micrometer range (nanoparticles) could support the findings regarding crystallite size reduction suggested via XRD (Figure 3B).

### 3.2. Conversion of Aged Biogenic Calcite into Phosphate Minerals

The four powders treated with phosphoric acid, following the stoichiometry for hydroxyapatite conversion, were generated under a careful drop-by-drop procedure to avoid pouring, an effervescent material which rapidly revealed under optical microscopy a typical morphology of brushite crystals growing on calcite particles. The conversion occurred instantly under ambient conditions, with the appearance of flower-like shaped bunches of elongated phosphate crystals seeded on calcite particles, growing rapidly in a matter of seconds. These details were observed under optical microscopy visualization of the Raman system. Thus, as a preliminary evaluation of the process, we used the comparative FT-Raman analyses of the starting material and of the conversion product. The reason for using FT-Raman with 1064 nm laser excitation instead of the micro-Raman system was the strong fluorescence of the conversion product under visible or 785 nm laser line excitation.

The transformation of the biogenic calcium carbonate powders into calcium phosphate involves an acid–base reaction between calcium carbonate and phosphoric acid, leading to partial dissolution of the carbonate matrix and reprecipitation of a calcium phosphate phase. During this process, calcium ions released from the solid react with phosphate species in solution, accompanied by the evolution of carbon dioxide gas. The overall transformation can be generally represented as follows:

CaCO_3(s)_ + H_3_PO_4(aq)_ + 2H_2_O_(l)_→CaHPO_4_⋅2H_2_O_(s)_ + CO_2(g)_↑

At room temperature, this reaction yields dicalcium phosphate dihydrate (brushite) as confirmed by XRD and Raman spectroscopy. The process proceeds through proton-assisted dissolution of the carbonate matrix, followed by precipitation of calcium phosphate phases. In addition, a recent, dedicated paper [32] investigated the conversion of biogenic carbonate into phosphate mineral using five pathways and showed that reaction parameters—including the calcite-to-phosphoric acid ratio, particle size, and thermal treatment—strongly influence phase composition and conversion efficiency. Stoichiometric reactions produced mixed calcium phosphate phases, while finer particles and excess acid improved conversion to monocalcium phosphate and organic matrix removal. Thermal treatment altered product composition: 700 °C yielded whitlockite-rich phases, and 1200 °C favored hydroxyapatite. Thus, here, we aimed to exploit these findings and go in favor of greener conversion with lower energy consumption, eliminating excess acid, thus favoring the brushite conversion, which is a stable form of hydrated calcium phosphate mineral, an attractive, industrially important final product resulting from long-term preserved crustacean waste.

#### 3.2.1. FT-Raman Analysis of Starting Material and Hydrothermal Conversion Product

**Figure 7:**
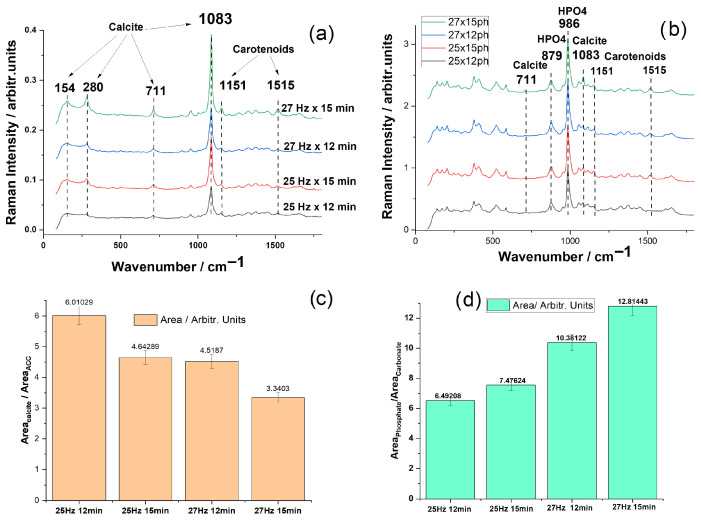
(**a**) Comparative FT-Raman spectra of aged crustacean powders obtained through ball milling with different milling times and frequencies; (**b**) spectra of the conversion products showing the dominant bands of brushite (CaHPO_4_⋅2H_2_O), residual (trace) calcite, and additional bands of carotenoids, as indicated; (**c**) the plotted ratio of the calculated band area calcite/ACC in the starting powders, and (**d**) ratio of the phosphate/carbonate in the final product (the band at 986 cm^−1^ of HPO_4_ and 1083 cm^−1^ of carbonate were used for calculation).

We compared the pigment and mineral Raman bands profile in non-resonance conditions, using the NIR excitation at 1064 nm (FT-Raman technique), which offered the advantage of low background and a reliable organic to inorganic band ratio, reflecting the non-resonance contribution of the pigments, mineral, and chitin to the overall Raman spectra of the bulk powders content. Further, the conversion product tracking was assessed using the starting powders as a reference signal to evaluate the involution of the carbonate Raman band in favor of the phosphate stretching mode increase. The recorded FT-Raman spectra for all four powder samples (Figure 7a) displayed, as expected, key peaks associated with the calcite phase of Mg-calcium carbonate in agreement with the micro-Raman spectra excited with 532 nm (Figure 5), but with the advantage of a much lower fluorescence background. The drawback of the NIR excitation is the low intensity of detected carotenoids; however, it is still relevant for the non-resonance excitation. Under NIR excitation at 1064 nm, the most intense Raman signal from powders is due to the carbonate mode at 1085 cm^−1^. Additional characteristic bands of calcite are clearly visible at 711 cm^−1^ (in-plane bending, ν_4_), as well as the lattice modes at 280 and 154 cm^−1^.

Following the phosphoric acid treatment, the reaction products were characterized using FT-Raman spectroscopy and micro-Raman with 785nm excitation. The FT-Raman spectra of the four conversion products (Figure 7b) displayed a combination of residual calcite signatures and distinct phosphate bands, aligning with the reference spectrum of brushite (CaHPO_4_⋅2H_2_O) from the RRUFF database (RRUFF ID: R070554).

The crystalline/amorphous ratio has been calculated from the ratio of the corresponding band areas from the FT-Raman spectra of the bulk powders. The deconvoluted bands are given in the Supplementary Figure S1, and the plot of the ratio of band areas is given in Figure 7c, suggesting that higher frequency and time of milling result in a higher amorphous phase. Amorphization by ball milling involves complex mechanisms to include mechanical impact creating defects, grain size dynamics, and kinetic competitions. The obtained results here demonstrate the amorphization of biogenic carbonate powder from crustaceans under calibrated ball milling, where time or frequency increase produced slightly different effects. However, from the currently applied paired values, the amorphization status clearly increased, according to the measured band area of the ACC component of the deconvoluted carbonate Raman band. Figure 7b displays the FT-Raman spectra of the reaction products between biogenic carbonate material and phosphoric acid, where bands corresponding to brushite (879 and 986 cm^−1^) and carotenoids (1151 and 1515 cm^−1^) are evident. The emergence of strong bands at 879 cm^−1^ (HPO_4_^2−^ bending) and 986 cm^−1^ (ν_1_ symmetric stretching of HPO_4_^2−^) corresponds well with the characteristic modes of brushite. The dominance of the phosphate bands in the FT-Raman spectrum of the conversion product confirmed the successful chemical transformation of carbonate material into a hydrated calcium phosphate phase. Still, remnant calcite bands at 711 cm^−1^ and 1083 cm^−1^ were observed, indicating incomplete reaction of the original biogenic material.

Under 785 nm excitation, still considerable background in the micro-Raman spectra of the conversion product is observed, but two weak bands, presumably attributable to traces of calcite and ACC, were noted in the spectra (Supplementary Figure S3).

Notably, the bands at 1515 and 1151 cm^−1^, assigned to carotenoids [16,21,32], highlighted the preservation of these important antioxidants during the reaction and demonstrated the occurrence of an “antioxidant-enriched phosphate mineral”, highlighting the biocarbonate origin of the starting material.

Carotenoid preservation post-conversion was unexpected and may open promising perspectives on the synthesis of attractive biomineral components for the bone substitutes field.

The FT-Raman spectra (Figure 7b) indicated the presence of all the Raman bands of brushite (CaHPO_4_·2H_2_O), with strong bands at 986 and 879 cm^−1^, along with other weaker bands at 585, 522, 413, 379, 206, and 176 cm^−1^. In addition, weaker, residual calcite bands are still observed, suggesting that a small amount of unreacted calcite remains. The carotenoid bands also appear in the final product, with relative intensities comparable to the strongest spectral bands, indicating that the carotenoids were preserved during the phosphoric acid treatment of biogenic powders.

The chitin characteristic bands, particularly those near 1656 cm^−1^ (amide I) and 2880 cm^−1^ (C–H stretching), remain detectable in the reaction product, although with lower intensity compared to the pure α-chitin spectrum (Figure S2, the Supplementary Materials). These bands are visible as small, weak peaks and show no noticeable shifts or intensity changes after conversion, confirming that the chitin framework remains structurally intact. Moreover, no additional Raman features associated with P–O–C or P–O–N linkages were observed, indicating that chitin was not phosphorylated under the applied conditions. The phosphate ions preferentially react with the calcium carbonate phase rather than with the chitinous organic matrix.

To assess the influence of milling time and frequency on the conversion of calcite to calcium phosphate, the intensities of the two main bands—986 cm^−1^ (HPO_4_^2−^) and 1083 cm^−1^ (CO_3_^2−^)—were measured for each sample. From the plot in Figure 7d, one can observe that, for the first three samples (25 Hz × 12 min, 25 Hz × 15 min, 27 Hz × 12 min), the conversion rate defined by the phosphate/carbonate band ratios is increasing as the milling time and the frequency increases (and powder particle size decreases), as expected, as lower powder particle sizes have a higher surface area available for reaction.

Overall, the conclusion is that brushite is the dominant crystalline phase formed from all tested powders, regardless of their size distribution. Slight variations in Raman band intensities across the four conversion products suggest differences in conversion efficiency and slightly different residual biogenic content, influenced by processing conditions.

#### 3.2.2. X-Ray Diffraction Analysis of Conversion Product

**Figure 8:**
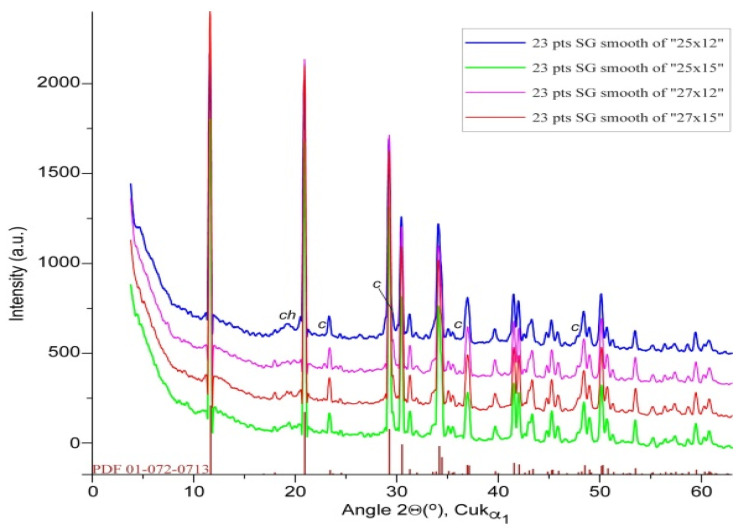
XRD patterns of the reaction product resulted from biogenic calcite powders after the phosphoric acid treatment, and the pattern of brushite (calcium hydrogen orthophosphate dihydrate) from PDF file 01-0720713 [30]. The marked peaks were attributed to chitin (ch) and unreacted calcite (c).

The structural transformation of biogenic calcite during its conversion into phosphate minerals is illustrated in the XRD profiles of the four products (Figure 8), with the nearly complete disappearance of calcite-specific peaks and the appearance of new reflections that matched well with the standard pattern of brushite (CaHPO_4_·2H_2_O), as indexed in the ICDD PDF2 database [30]. The formation of brushite across all samples suggests an efficient ion-exchange process under the applied reaction conditions, where calcium ions from calcite reacted with phosphate species to yield a stable calcium phosphate phase [29,30]. Notably, the newly formed brushite has clear and sharp diffraction features, implying that the conversion proceeded without significant amorphization. This preservation of crystalline order throughout the transformation process reflects a controlled reaction mechanism that maintains structural integrity, which is particularly advantageous for applications requiring defined crystallinity, such as in bone grafting or bioactive material development. These findings demonstrate the potential of aged biogenic calcite waste as a valuable precursor for producing functional phosphate-based biomaterials through processing routes in line with blue bioeconomy concepts.

#### 3.2.3. SEM-EDX Analyses of the Resulted Phosphate Mineral

**Figure 9:**
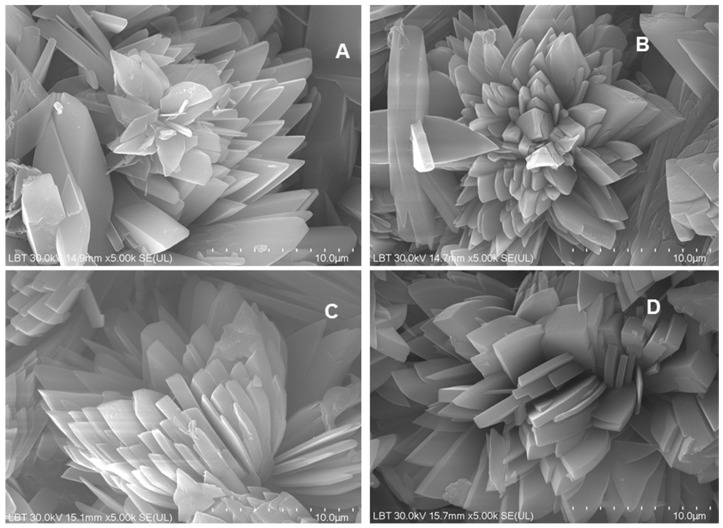
SEM images recorded from the phosphate mineral (brushite) resulted from the conversion reaction of the four biogenic powders: (**A**) 25 Hz_12 min; (**B**) 25 Hz_15 min; (**C**) 27 Hz_12 min; (**D**) 27 Hz_15 min. Scale bar: 10 µm.

Scanning electron microscopy (SEM) was used to examine the morphology of the phosphate crystals obtained from the conversion of biogenic powders milled under different conditions. All samples exhibited characteristic dicalcium phosphate dihydrate (brushite CaHPO_4_·2H_2_O) crystal habitus, appearing as flower-like (Figure 9), radially oriented (commonly after the (010) plane) monoclinic-prismatic crystals elongated on the c-axis, with well-developed (100), (010), and (001) faces, the (101) type occurring only sporadically. The individual crystals ranged, on average, from 1 to 10 µm long, with a thickness ranging between 0.3 and 2 µm, forming dense and layered textures visible across all four samples. No statistically significant morphological differences were observed among the four samples, suggesting that brushite formation was consistent regardless of the milling parameters.

**Figure 10:**
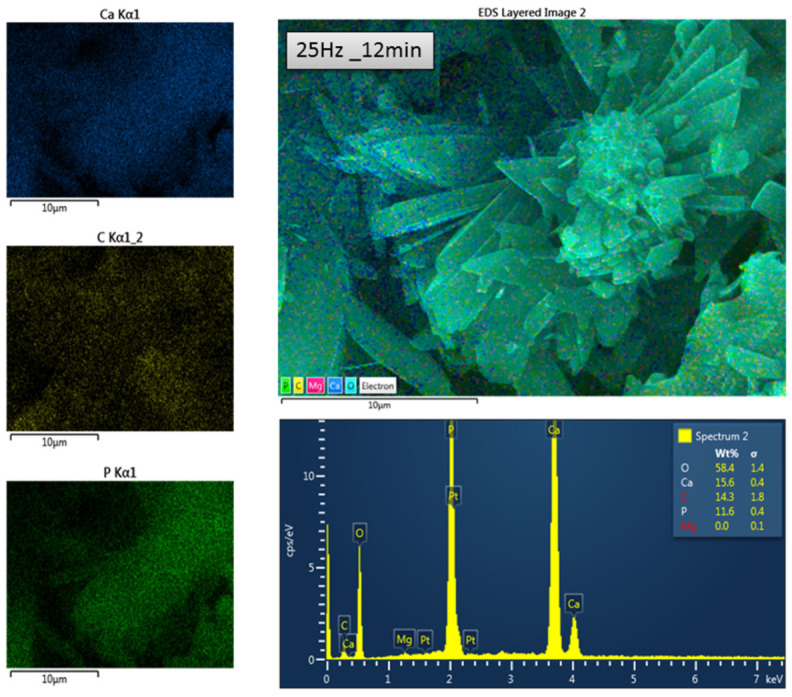
EDX spectrum and maps of elements distribution: overlay (large map) and individual P, Ca, and C (maps on the left), over the surface morphology of the phosphate mineral (brushite) resulted from the biogenic powder batch (25 Hz_12 min).

Energy dispersive X-ray spectroscopy (EDX) confirmed the presence and spatial distribution of calcium (Ca), phosphorus (P), and oxygen (O), consistent with the brushite composition already determined by XRD, as shown, for example, in the brushite resulting from the sample 25 Hz_12 min in Figure 10. Elemental distribution mapping showed, however, a slightly different ratio of Ca and P from 1:1 throughout the mineral phase, with minimal residual carbon, indicating different conversion efficiency from biogenic calcite to calcium phosphate. A comparative analysis of elemental ratios at different milling frequencies further supports these findings, as shown in Supplementary Figure S4, which illustrates the variations in Ca:P and Ca:C ratios. These microstructural and compositional analyses reinforce the successful transformation pathway, although incomplete for the chosen baseline reaction conditions, suggesting that each calibrated milling parameters need further optimization of the conversion reaction for the desired phosphate mineral. In this respect, a dedicated study has been recently published [32].

For example, the elemental distribution maps of one batch corresponding to the carbonate powder milled under 25 Hz_12 min) showed in Figure 10, the resulting mineral indicated that the elemental composition is dominated by oxygen (58.4 wt%), with notable contributions from calcium (15.6 wt%), carbon (14.3 wt%), and phosphorus (11.6 wt%), while magnesium was detected only at a negligible level within the error margin. The semiquantitative EDX results for the phosphate conversion products are summarized in Table 2, showing the elemental composition and corresponding standard deviations for each batch The highest P:Ca ratio of 74.3% for the given reaction conditions has been reached for the batch (25 Hz_12 min), followed by 67.5% for (25 Hz_15 min), and 58% and 59% for the batches (27 Hz_12 min and 27 Hz_15 min), respectively. They are shown comparatively in the Supplementary Figure S4. These results, although semiquantitative, are due to the limitations of the technique for random micro-surfaces; however, they are validated by the Raman spectroscopy results (Supplementary Figure S3), stating the highest intensity brushite peaks in the spectra recorded on phosphate minerals from the same carbonate batch. Thus, it is suggested that excessive ball milling would not necessarily result in a better conversion ratio, rather for each batch an optimal time–frequency set would be correlated with the optimized reaction conditions.

Chitin is not the major constituent of the biogenic material, as the XRD, IR, and Raman spectroscopy consistently confirmed calcite as the dominant phase, with chitin signals appearing much weaker. Consequently, the nitrogen associated with chitin structure falls below the detection limit of the EDX technique.

Although the conversion of biogenic calcium carbonate to phosphate minerals is apparently focused on targeting hydroxyapatite product for biomedical purposes [32,33,34,35,36] as a potent synthetic bone graft substitute, reaching this phase of phosphate mineral is conditioned by high energy consumption for necessary heating up to 900–1200 °C [32,33], and taking into account the species-specific properties determined by the starting material provenance. For example, biogenic carbonate derived from bivalves with polymorphism being species-specific, comprising various aragonite/calcite/ACC balance, the conversion route to phosphate minerals varied substantially. Adding here the specific organic scaffold, it clearly influences the conversion conditions and discriminates them from geogenic calcium carbonate [37] or from other biogenic sources. The present results highlighted the advantage of crustacean-derived biogenic calcite over other reported species due to the naturally embedded astaxanthin to further act as a co-existent antioxidant in phosphate composites.

## 4. Conclusions

This study reports for the first time the effects of calibrated ball milling of aged biogenic carbonate materials derived from crustacean shells, as a complex mineral–organic framework, where the expected organic counterpart degradation in aged material, particularly of the carotenoids in the intricate 3D porous structure, was unexpectedly absent. Advanced characterization techniques, including Raman spectroscopy, FT-IR, XRD, DLS, and SEM-EDX, were employed to analyze controlled milling-induced transformations. DLS analysis revealed a broad size distribution in milled samples, with sedimentation effects influencing measurement reliability. This research highlights the potential of ball-milled crab shell carbonate as a sustainable material for environmental and biomedical applications while addressing challenges related to crystallinity, porosity, and preservation of the organic components’ identity during frequency and time change in ball milling settings. The findings contribute to a broader understanding of the mechanical processing effects on biogenic materials, offering insights for optimizing their reuse according to the resulting powder’s purpose.

We provided valuable insights into the effects of slightly different ball milling conditions on the structural and functional properties of aged biogenic carbonate from crustacean waste crab shells. Slight variations in milling parameters, including frequency and duration, induced subtle changes in the particle size distribution, indicating that the calibrated milling conditions impact on particle size and functionality control. The Raman, FT-IR, and XRD analyses revealed that the primary crystalline phase of calcium carbonate, primarily in the form of calcite, remained stable across all milling conditions, accompanied by subtle amorphization. The presence of astaxanthin, a natural pigment in the crab shells, was confirmed via Raman spectroscopy, with no significant degradation or structural alteration observed in aged material during milling. Unexpectedly, carotenoid was still detectable even in the reaction products, provided that the excess acid reaction condition was eliminated.

Across four powder batches produced under distinct milling frequencies and durations, dynamic light scattering confirmed only subtle particle size variation, while Raman spectroscopy, XRD, FT-IR, and SEM-EDX confirmed structural and chemical integrity and highlighted the subtle amorphization induced by slightly different milling parameters, which, in turn, drove slightly different conversion efficiency into phosphate mineral. Strikingly, all powders underwent rapid transformation into dicalcium phosphate dihydrate (brushite) enriched with carotenoids upon reaction with phosphoric acid. This work reveals, for the first time, that years-aged biogenic Mg-calcite waste not only preserves its naturally embedded carotenoids but also offers a direct route to functional phosphate composites, establishing its untapped value in environmental and biomedical applications.

## Figures and Tables

**Table 1:** Averaged particle size optically observed in powders at different magnifications (5×, 20×, 50×, and 100×) for each milling condition (25 Hz for 12 min, 25 Hz for 15 min, 27 Hz for 12 min, and 27 Hz for 15 min). Values are given in micrometers (μm). Scale bars: 200, 50, 20, 10 μm.

Milling Conditions (Frequency, Time)	Particle Size Observed Under Optical Microscopy/μm			
	5× Obj.	20× Obj.	50× Obj.	100× Obj.
25 Hz, 12 min	10–400	>5	2–40	~2–20
25 Hz, 15 min	10–300	>5	2–30	~1–15
27 Hz, 12 min	10–300	>5	2–30	~1–15
27 Hz, 15 min	10–300	>5	2–30	~1–15

**Table 2:** Summarized data resulted from the three independent data sets of the EDX semiquantitative analysis of the phosphate conversion products for the investigated powder batches and their conversion output. The highest P:Ca ratio of 74.3% for the given reaction conditions has been reached for the batch (25Hz_15 min).

	Wt% (25 Hz_12 min)	σ	Wt% (25 Hz_15 min)	σ	Wt% (27 Hz_12 min)	σ	Wt% (27 Hz_15 min)	σ
O	58.4	1.4	58.9	1.4	53.6	1.8	54.6	1.5
Ca	15.6	0.4	18.8	0.5	20.0	0.7	15.8	0.5
C	14.3	1.8	9.7	1.8	14.8	2.3	20.3	1.8
P	11.6	0.4	12.7	0.4	11.6	0.5	9.3	0.3
Mg	0.0	0.1	-	-	-	-	-	-

## Data Availability

The original contributions presented in this study are included in the article/Supplementary Material. Further inquiries can be directed to the first and corresponding author.

## Supplementary Materials

The following supporting information can be downloaded at: https://www.mdpi.com/article/10.3390/ma18225119/s1, Figure S1: carbonate stretching mode from the FT-Raman spectra of biogenic powders obtained under different milling times and frequencies, as indicated, and their comparative analysis of crystalline/amorphous ratio from band profile. Deconvolution clearly indicated the ACC and crystalline counterpart in each sample. Figure S2: comparative display of the FT-Raman spectra of starting powder and reaction product illustrating the co-existence of minor, unchanged chitin bands. Alpha-chitin reference spectrum is given in red line. Figure S3: Left: micro-Raman spectra (average of 20 points acquisition, background subtracted) of phosphate-converted biogenic powders previously milled under different conditions, as indicated by color codes: 25 Hz for 12 min, 25 Hz for 15 min, 27 Hz for 12 min, and 27 Hz for 15 min. The reference Raman spectrum of brushite RRUFF ID: RO70554 recorded with 785 nm excitation (unoriented) is shown in the top. Right: overlayed spectra, highlighting the 100–1600 cm^−1^ range. Figure S4: comparison of the phosphorus-to-calcium and calcium-to-carbon ratios calculated from the EDX data of the reaction product (brushite), corresponding to the four batches milled under 25 Hz for 12 min, 25 Hz for 15 min, 27 Hz for 12 min, and 27 Hz for 15 min, as shown in the sample name.

## Abbreviations

The following abbreviations are used in this manuscript:

FT-IR	Fourier Transform Infrared Spectroscopy
DLS	Dynamic Light Scattering
SEM-EDX	Scanning Electron Microscopy–Energy Dispersive X-ray Spectroscopy
