# Supplementary material for: Aged Biogenic Carbonates from Crustacean Waste: Structural and Functional Evaluation of Calibrated Fine Powders and Their Conversion into Phosphate Minerals
Source: Materials (Basel). 2025 Nov 11;18(22):5119. doi: 10.3390/ma18225119 (PMC12654126; doi:10.3390/ma18225119)
Supplement: Supplementary file 1 [file materials-18-05119-s001.zip › materials-3861653-supplementary.pdf]

Supplementary Materials

to

Article

Aged Biogenic Carbonates from Crustacean Waste: Structural and Functional Evaluation of Calibrated Fine Powders and Their Conversion into Phosphate Minerals

Ilirjana Bajama [1,2,*], Karlo Maškarić [1,2,3], Geza Lazar [1,2], Tudor Tamaş [4], Codruţ Costinaş [1,2], Lucian Barbu-Tudoran [4,5] and Simona Cîntă Pinzaru [1,2,*]

[1] Faculty of Physics, Babes-Bolyai University, Kogalniceanu 1, 400084 Cluj-Napoca, Romania; karlo.maskaric@ubbcluj.ro (K.M.); geza.lazar@ubbcluj.ro (G.L.); codrut.costinas@ubbcluj.ro (C.C.)
[2] Institute for Research, Development and Innovation in Applied Natural Science, Babes-Bolyai University, Fântânele 30, 400327 Cluj-Napoca, Romania
[3] Advanced Research and Technology Center for Alternative Energy, National Institute for Research and Development of Isotopic and Molecular Technologies, Donath 67-103, 400293 Cluj-Napoca, Romania
[4] Faculty of Biology and Geology, Babes-Bolyai University, Kogalniceanu 1, 400084 Cluj-Napoca, Romania; tudor.tamas@ubbcluj.ro (T.T.); lucian.barbu@ubbcluj.ro (L.B.-T.)
[5] Integrated Laboratory of Electron Microscopy, National Institute for Research and Development of Isotopic and Molecular Technologies, Donath 67-103, 400293 Cluj-Napoca, Romania
* Correspondence: ilirjana.bajama@ubbcluj.ro (I.B.); simona.pinzaru@ubbcluj.ro (S.C.P.)

This material comprises four figures:

Figure S1: carbonate stretching mode from the FT-Raman spectra of biogenic powders obtained under different milling times and frequencies, as indicated, and their comparative analysis of crystalline/amorphous ratio from band profile. Deconvolution clearly indicated the ACC and crystalline counterpart in each sample.

Figure S2: comparative display of the FT-Raman spectra of starting powder and reaction product illustrating the co-existence of minor, unchanged chitin bands. Alpha-chitin reference spectrum is given in red line.

Figure S3: Left: micro-Raman spectra (average of 20 points acquisition, background subtracted) of phosphate-converted biogenic powders previously milled under different conditions, as indicated by color codes: 25 Hz for 12 min, 25 Hz for 15 min, 27 Hz for 12 min, and 27 Hz for 15 min. The reference Raman spectrum of brushite

RRUFF ID: RO70554 recorded with 785 nm excitation (unoriented) is shown in the top. Right: overlayed spectra, highlighting the 100–1600 cm^{-1} range.

Figure S4: comparison of the phosphorus-to-calcium and calcium-to-carbon ratios calculated from the EDX data of the reaction product (brushite), corresponding to the four batches milled under 25 Hz for 12 min, 25 Hz for 15 min, 27 Hz for 12 min, and 27 Hz for 15 min, as shown in the sample name.

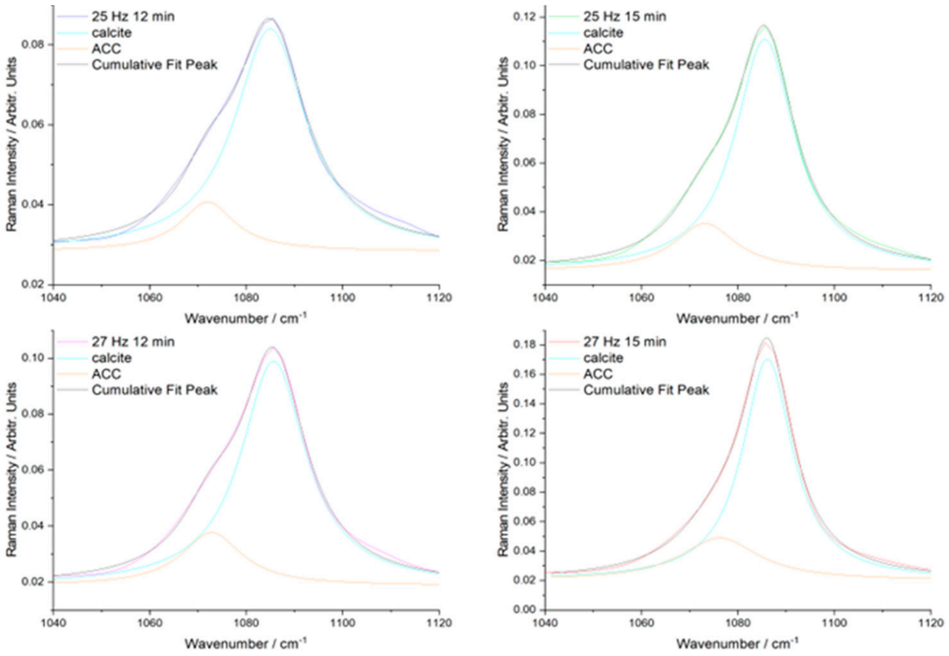

Figure S1. carbonate stretching mode from the FT-Raman spectra of biogenic powders obtained under different milling times and frequencies, as indicated, and their comparative analysis of crystalline/amorphous ratio from band profile. Deconvolution clearly indicated the ACC and crystalline counterpart in each sample.

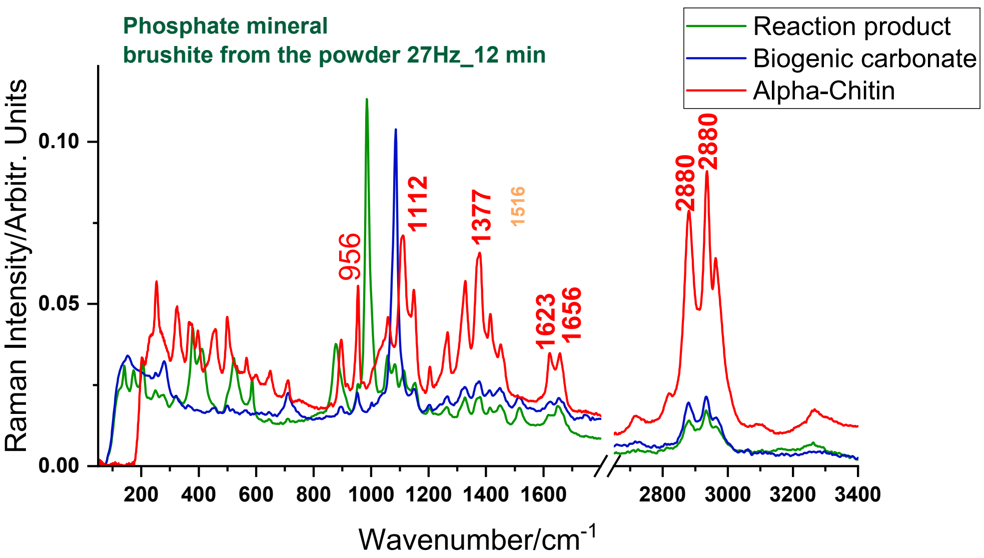

Figure S2. comparative display of the FT-Raman spectra of starting powder and reaction product illustrating the co-existence of minor, unchanged chitin bands. Alpha-chitin reference spectrum is given in red line.

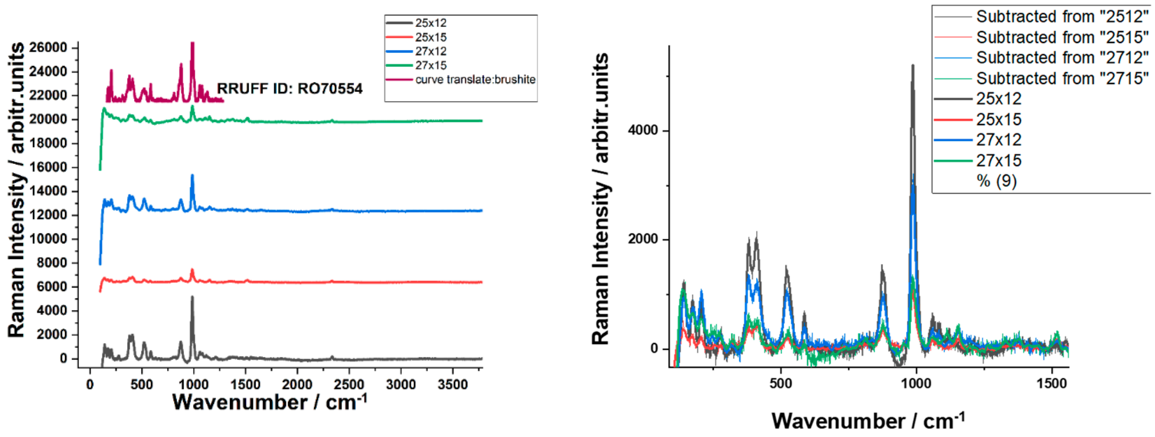

Figure S3. Left: micro-Raman spectra (average of 20 points acquisition, background subtracted) of phosphate-converted biogenic powders previously milled under different conditions, as indicated by color codes: 25 Hz for 12 min, 25 Hz for 15 min, 27 Hz for 12 min, and 27 Hz for 15 min. The reference Raman spectrum of brushite RRUFF ID: RO70554 recorded with 785 nm excitation (unoriented) is shown in the top. Right: overlayed spectra, highlighting the 100–1600 cm^{-1} range.

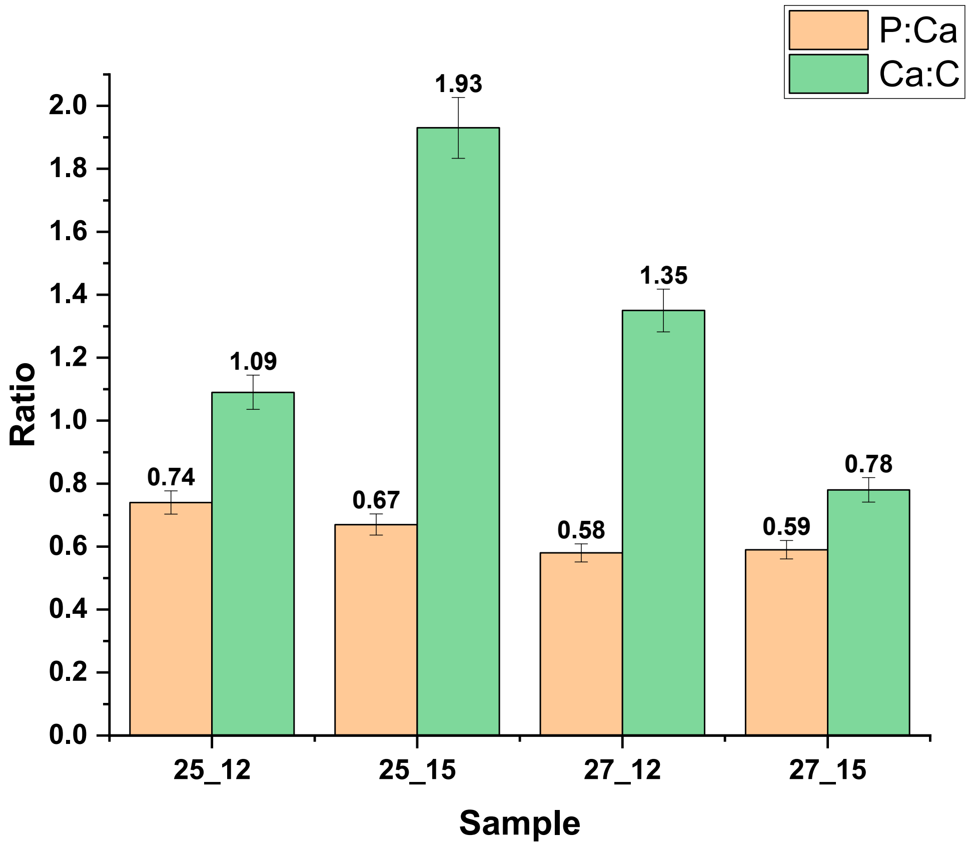

Figure S4. comparison of the phosphorus-to-calcium and calcium-to-carbon ratios calculated from the EDX data of the reaction product (brushite), corresponding to the four batches milled under 25 Hz for 12 min, 25 Hz for 15 min, 27 Hz for 12 min, and 27 Hz for 15 min, as shown in the sample name.
